# Identifying the role of public health nurses during first postnatal visits: Experiences of mothers and public health nurses in Ireland

**DOI:** 10.1016/j.ijnsa.2020.100017

**Published:** 2021-01-05

**Authors:** Martina Giltenane, Ann Sheridan, Thilo Kroll, Kate Frazer

**Affiliations:** a Nursing and Midwifery Planning and Development Unit, Health Service Executive, Swords Business Campus, Balheary Road, Swords Co. Dublin, Ireland; b Lecturer and Researcher Mental Health. Subject Head - Mental Health Nursing, Chairperson Irish Institute of Mental Health Nursing, UCD School of Nursing, Midwifery & Health Systems, Room B310 UCD Health Sciences Centre, University College Dublin, Belfield, Dublin 4, Ireland; c Professor of Health Systems Management, Associate Dean for Research, Innovation and Impact, UCD School of Nursing, Midwifery and Health Systems, Fellow at the UCD Geary Institute for Public Policy, Room B225 UCD Health Sciences Centre, University College Dublin (UCD), Belfield, Dublin 4, Ireland; d Director Graduate Research, Head of Subject: Public Health and Community Nursing, Fellow European Academy Nursing Science, Fellow UCD Geary Institute Public Policy, Room B224, UCD School of Nursing, Midwifery and Health Systems, UCD College of Health and Agricultural Sciences, Belfield, Dublin 4, Ireland

**Keywords:** Empowerment, Health Promotion, Mothers, Postnatal visit, Public Health Nurse, Relationship-Building, Qualitative exploration

## Abstract

**Aim and Objective:**

Explore the views and experiences of quality of care provided during a first postnatal visit from the perspectives of mothers and public health nurses.

**Background:**

Quality of nursing care is high on the policy agenda internationally, especially in the year of Nurse and Midwife. Public health nurses are acknowledged nationally and globally as essential health professionals supporting infants and parents. The first postnatal visit provided by the public health nurse is a complex intervention for mothers incorporating physical, social, educational and emotional support, and we know little about the quality of care provided. International evidence suggests a lack of consensus in setting priorities for this crucial visit.

**Design:**

Exploratory, qualitative design, utilising focus groups and interviews and analysed using thematic analysis.

**Setting/Participants:**

Nineteen public health nurses from all four health service regions and five mothers from one health service region were interviewed to explore the quality of care provided during first postnatal visits.

**Methods:**

Adopting qualitative data collection methods for the study; five individual semi-structured interviews with mothers ascertained their experiences of care. Further, four focus groups with public health nurses explored their understanding of the quality of care provided to mothers. The data collection period was August 2015 to January 2016. Interviews were audio-recorded, transcribed verbatim and analysed using thematic analysis.

**Results:**

Mothers and public health nurses identified that public health nurses were crucial for providing support during the first postnatal visit as mothers required care and advice around physical, psychological and social wellbeing for themselves and their new infant. Discordant experiences of quality care and lack of standardisation of care practices emerged. Nurses identified relationship building, empowerment and health promotion as pivotal to the public health nurses' role. Mothers acknowledged the supportive and practical aspects of the care provided.

**Conclusion:**

This is the first Irish study combining nurses and mothers experiences, identifying care provided at the first postnatal visit, presenting the quality of care experienced. This visit is vital for relationship-building and empowering mothers' child-care and self-care abilities. The findings provide an insight into how quality of care is perceived by public health nurses and mothers. Experiences facilitate reflection and the development of quality improvements to ensure mothers and infants are adequately supported during the first postnatal visit.

**Contribution of the Paper**

Examining the perceived quality of care provided by public health nurses at their first postnatal visits is crucially linked to child and maternal health outcomes. This study defines the quality of practice from mothers’ and public health nurses’ perspectives, revealing consistent as well as different experiences.

**What is already known about the topic?**

• The first postnatal visit is an essential professional visit by Public Health Nurses with a mother and her infant.
• There is limited evidence about the quality of care provided during this interaction from a service user or professional perspective.

**What this paper adds?**

• Relationship building is an essential component of the first postnatal visit, and mothers value the supportive and practical elements.
• The priorities in care differed for mothers and nurses.
• A lack of standardisation exists and results in variation in the experience of quality care.
• This study identifies indicators of measurable postnatal care.

## 1 Introduction and background

Care in the postnatal period, promoting optimal social, physical and emotional health and wellbeing of the mother and baby within the family and community is core to supporting women in the transition to motherhood and is recognised internationally (Danbjorg et al., 2014, Haran et al., 2014, Martin et al., 2014, National Institute for Clinical Excellence (NICE), 2015, HSE, 2015, Dahlberg et al., 2016, Giltenane et al., 2016b, Giltenane et al., 2016a, Bagheri et al., 2017, Helsloot et al., 2017, Probandari et al., 2017, Slomian et al., 2017, Walker et al., 2019). Where the practice exists internationally, it is undertaken by public health nurses (PHN), community midwives or health visitors. The time frame for engaging with women at home after birth as well as the professional expertise varies globally ranging from within one day following discharge from the maternity unit in the United Kingdom (UK) by community midwives and within 72 hours in Ireland by PHNs (Stewart-Moore et al., 2012, Haran et al., 2014, NICE, 2015, HSE, 2015, Dahlberg et al., 2016, Giltenane et al., 2016a, Giltenane et al., 2016b, HSE, 2017). PHNs in Ireland may also possess a midwifery qualification. In countries including Ireland, the UK and Norway discharge of mothers and infants from the hospital can be six to eight hours postpartum; thus the importance of the first postnatal visit has increased in significance (Leahy-Warren, 2005, O'Dwyer, 2009, Haran et al., 2014, Dahlberg et al., 2016). The NICE (2015) postnatal guideline provides a comprehensive framework to assess mothers during a first postnatal visit in the UK. No national postnatal guideline exists in Ireland; a few ad-hoc local guidelines are available. While the first postnatal visit is acknowledged as central to establishing rapport (Appleton and Cowley, 2008, Leahy-Warren, 2005, Phelan, 2014, Dahlberg et al., 2016), what constitutes this visit to meet the needs of a mother and her infant is undefined and unfortunately limited to certain contexts internationally (Phelan, 2014, Mcleish et al., 2019).

International evidence identifies the enormity of challenges in becoming a parent and encompasses physical, psychological and practical adaptation to motherhood (Appleton and Cowley, 2008, Leahy-Warren, 2005, Phelan, 2014, Dahlberg et al., 2016, Walker et al., 2019). However, recommendations such as NICE guidelines, do not provide evidence of the quality of care provided.

While there is no definitive definition of quality care, it includes patient safety, patient experience and effectiveness of care (UK's Department of Health, 2008, World Health Organisation, (WHO) 2008, Lillrank, 2015). Community engagement is an integral component of improving the quality of care for mothers and newborns (Tunçalp et al., 2015, WHO, 2016). Quality care for mothers and newborns in communities requires competent health professionals and the availability of essential resources (Tunçalp et al., 2015, WHO, 2016). Actionable information systems where record keeping enables review and audit mechanisms should be available together with functional referral systems between levels of care. Mothers’ experience of care should include effective communication, respect for their expectations and their rights (WHO, 2016). Care should be delivered with respect and dignity and mothers should have access to the social and emotional support of her choice (Tunçalp et al., 2015, WHO, 2016). The current study explored PHNs’ and mothers’ experiences of the quality of care provided in order to identify key indicators of care.

## 2 Supporting transition to motherhood

Recognition that the transition to motherhood can involve some degree of crisis or difficulty enhances the importance for postpartum support by community midwives, health visitors and PHNs (Leahy-Warren, 2005, Appleton and Cowley, 2008, Haycock-Stuart and Kean, 2012, Garvan, 2016, Mcleish et al., 2019, Wayman, 2019). There is general agreement that sufficient time is necessary to listen and support parents and to develop a partnership approach to supporting the mother and the infant (Appleton and Cowley, 2008, Leahy-Warren, 2005, O'Dwyer, 2009, Phelan, 2014, Dahlberg et al., 2016, Helsloot et al., 2017).

The visit ideally provides open communication and information sharing, which is critical to reduce anxiety and provide reassurance for mothers. It can also minimise maternal fatigue and stress, and enhance maternal coping capacity (Leahy-Warren, 2005, Byrd, 2006, Phelan, 2014).

Secondly, it enables review of the infant establishing wellness and validating the mothering abilities (Byrd, 2006, Stewart-Moore et al., 2012, Phelan, 2014, Dahlberg et al., 2016). Observation of interactions between a mother and her baby by health professionals provides essential affirming of the parental role (Kurth et al., 2016) during this transition.

## 3 Quality of care provided

Growing evidence suggests mothers’ priorities for postpartum care differ (Schmied and Bick, 2014, Phelan, 2014, Kurth et al., 2016, Monsen et al., 2017) as do mothers’ and nurses’ priorities for postpartum care and education (Newburn, 2010, Razurel et al., 2011, Barimani and Vickstrom, 2015, Cowley et al., 2018). Tailoring care is a means of improving the quality of care and outcomes for mothers and babies, and is essential in building a partnership (Monsen et al., 2017, Mcleish et al., 2019). PHNs, community midwives and health visitors are required to provide a standardised service while adapting care practices to meet individual needs (Phelan, 2014, Mcleish, 2019). Mothers expect professionals to be knowledgeable, skilful, flexible and observant to meet their needs (Kurth et al., 2016, Cowley et al., 2018). Quality of nursing care is high on the policy agenda internationally, especially in the year of Nurse and Midwife (International Council of Nurses, 2019) and is vital for continued improvement in community nursing practice (O'Dwyer, 2009, Haycock-Stuart and Kean, 2012, Helsloot et al., 2017). However, PHN and health visiting professional practices vary and a lack of consistency exists (Haycock-Stuart and Kean, 2012, McDonald et al., 2013, Giltenane et al., 2015, Giltenane et al., 2016a, Giltenane et al., 2016b, Noonan et al., 2016, Helsloot et al., 2017). This study aimed to explore the views and experiences of quality of care provided during a first postnatal visit representing perspectives of both mothers and nurses.

## 4 Methods

### 4.1 Research question

The research question guiding this study was: What was the quality of care delivery during a first postnatal visit that can guide improvements in the care provided to mothers and their infants?

### 4.2 Study design

An exploratory qualitative design was used with reporting guided by the consolidated criteria for reporting qualitative research (COREQ) (Tong et al., 2007). Qualitative methods were chosen because this was the best way to capture participants’ experiences (Creswell and Plano Clark, 2011) and gain rich and complete descriptions that could not have been captured in the quantitative arena (Patton, 2002, Creswell and Plano Clark, 2011). Focus groups were conducted with PHNs to understand the experiences of providing quality care and individual interviews with mothers to capture personal experiences (Creswell and Plano Clark, 2011). Krueger and Casey, (2015) identify that focus group interviews have been helpful in developing and maintaining quality improvement efforts in the past. These quality efforts have depended on widespread involvement, open communications, feedback and a nonthreatening environment. Due to the inherent nature of questioning of some sensitive information with mothers, individual semi-structured interviews were deemed more appropriate than focus groups for these participants (Krueger and Casey, 2015). Individual semi-structured interview format was to facilitate the collection of in-depth data that acknowledges the unique views and experiences of participants (Polit and Beck, 2013).

### 4.3 Participants sample

A purposive sample of PHNs and mothers, meeting the inclusion criteria (Table 1) participated. Strict inclusion and exclusion criteria was enacted to target participants who were best placed to discuss their views and experience in order to meaningfully contribute. PHNs and Director/Assistant Directors of Public Health Nursing were included as they were best placed to identify the current PHN practices. Mothers between six weeks and six months from discharge were chosen. The challenge for mothers was the time frame for inclusion; they may not wish to discuss care from PHNs at an early stage postpartum. However, the inclusion of mothers at this time point was to reduce recall bias. Fathers/Partners were not included as they may not be present at the time of the postnatal visit. In keeping with recommended guidelines in relation to appropriate numbers of focus group interviews, four focus group interviews comprising a total of 19 PHNs were carried out (Krueger and Casey, 2000, Rabiee, 2004). Five individual interviews were completed with mothers until data saturation was achieved (Kvale, 1996).

**Table 1:** Inclusion/exclusion criteria.

**Objective:** To explore PHNs perceptions about the quality of postnatal care for mothers during the first postnatal visit.		

Inclusion Criteria; • PHN actively caring for Mothers and their babies in the community in Ireland • Director of PHN or Assistant Director of PHN currently managing a PHN service in Ireland		Exclusion Criteria; • Community Registered General Nurses in Ireland as they do not hold a child and maternal caseload • PHN that is not actively caring for Mothers and their babies in the community in Ireland • Director of PHN or Assistant Director of PHN not currently managing a PHN service in Ireland

**Objective:** To explore mothers' perceptions and experiences of the quality of care provided by public health nurses at the first postnatal visit.		

Inclusion Criteria; • Women discharged from maternity services to the PHN service in the previous six weeks to six months period and residing in one Health Board region.	Exclusion Criteria; • Women discharged from maternity services to the PHN service not living in the specified Health Board region. • Fathers/Partners of women with children as they may not have been present at the first postnatal visit	

### 4.4 Recruitment

A third-party gatekeeper disseminated information about the study and facilitated recruitment and participation including Directors of Public Health Nursing who assisted in accessing and recruitment of nurses nationally. Recruitment of mothers was via liaising directly with general practitioners (family doctor) and with lactation consultants for breastfeeding support groups. Breastfeeding groups were chosen as a means of recruitment as groups of mothers attend within the early postnatal period. Formula feeding groups did not exist in the region at the time of recruitment and these mothers were captured via general practitioners. Gatekeepers displayed posters advertising the study which included instructions and the researcher's contact details should mothers wish to take part in this study. Participants contacted the researcher by telephone expressing interest to take part in this study. Participants provided their postal address and the researcher posted an introductory letter, an information sheet, an informed consent form and a stamped addressed envelope to return the signed consent to the researcher. Eight mothers expressed interest, five of which returned the consent forms and took part in the study.

### 4.5 Data collection

The data collection period was August 2015 to January 2016. One focus group was held in all four regional health boards in Ireland with between three and six participants in each group. The location for each was a primary care centre, and the duration of each focus group meeting was 55–105 minutes.

Interviews were conducted with mothers in neutral venues such as a quite space in a hotel lobby that accommodated mothers and babies which were selected by participants. The duration of the interviews was 20–30 minutes. Examples of semi-structured interviews and focus group topic guides (pilot tested in advance) are available in supplementary appendices 1 and 2.

## 5 Data management and analysis

### 5.1 Validity and reliability/rigour

All interviews and focus groups were audio-recorded with participants’ written consent, transcribed verbatim and findings are supported by direct quotations to allow the reader to judge the dependability. A random selection comprising 25% of the transcripts were reviewed and verified by an independent second coder using the coding framework (Burla et al., 2008). The coding framework was based on theoretical perspectives guided by the research objectives and also based on issues arising from the data (Attride-Stirling, 2001, Ezzy, 2013). There was a 74% agreement between data coders indicating a reasonable degree of consistency or 'reliability' (Julien, 2011). Julien et al. (2011) proposes acceptable reliability of a minimum of 60% agreement between qualitative data coders.

Data were de-identified using codes and pseudonyms. Thematic analysis was used and produced thematic networks; ‘web-like illustrations that summarise the main themes constituting a piece of text’ (Attride-Stirling, 2001, p.386). Six main steps to analysis were conducted; coding the material, identifying themes, constructing the networks, describing and exploring thematic networks, summarising thematic networks and interpreting patterns as outlined by Attride-Stirling (2001).

PHNs’ focus group data and mothers’ interview data were analysed in isolation before they were combined with equal weighting and presented in an integrated fashion. This enhanced the findings, prevents duplication and ensures the voices of service users were heard. This process of combining the focus group and individual interview findings enhances data richness. It is recognised as an effective means of strengthening the trustworthiness of data given that professionals’ and mothers’ views may differ (Shenton, 2004, Lambert and Loiselle, 2008, Hasson et al., 2019). Nurses’ views were verified against mothers’ and vice versa producing a picture of the attitudes, needs and behaviours of PHNs based on the contributions of PHNs and mothers (Shenton, 2004).

**Fig. 1:**
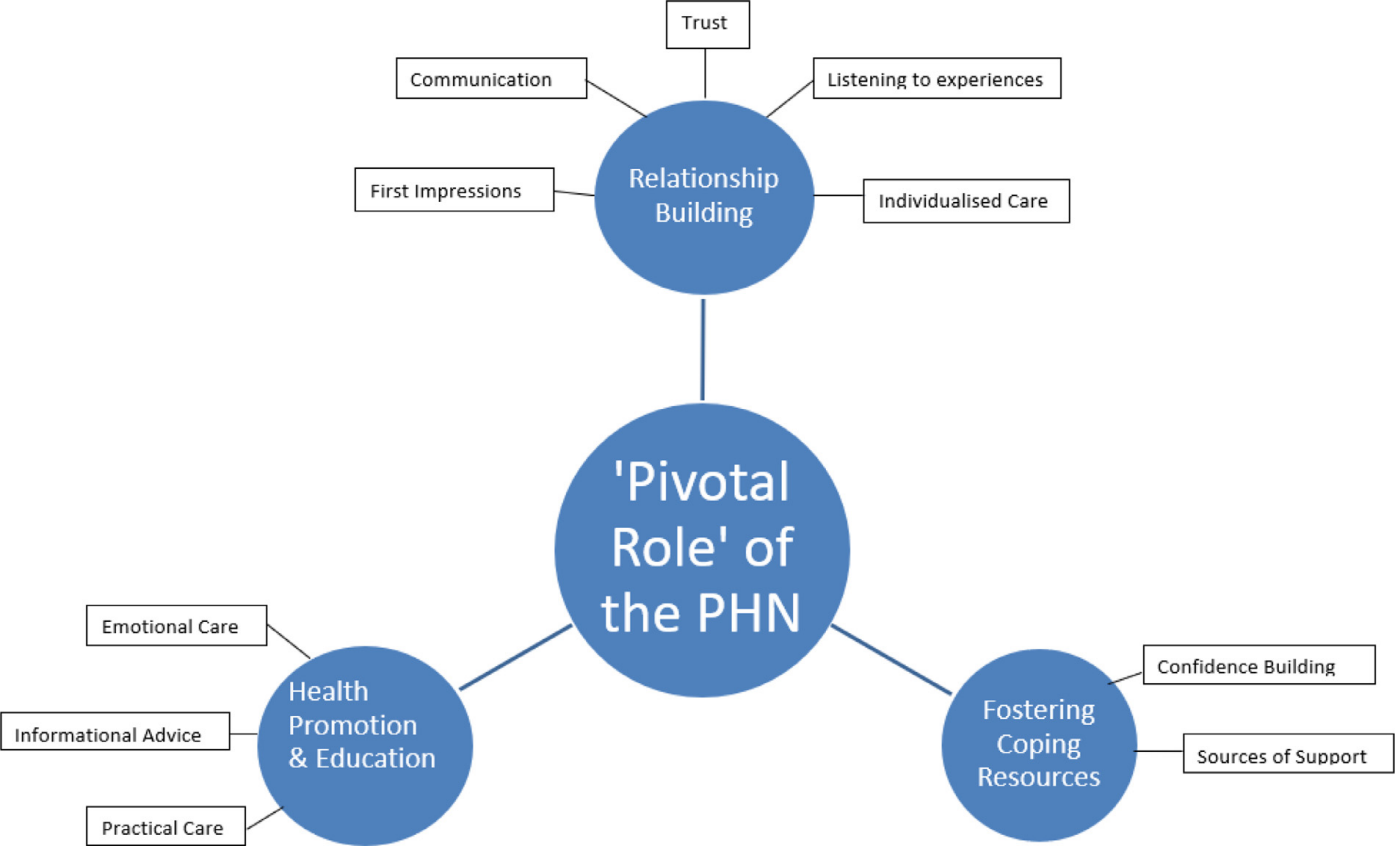
Global network map-the pivotal role of the public health nurse.

Examples of moving from codes to basic themes, moving from basic to organising themes and moving from organising themes to the global theme are presented in Tables 2 and 3. Thematic networks organising themes (Attride-Stirling, 2001) drawing on core features are common to qualitative approaches (Creswell et al., 2007) and assisted the process through graphical illustration, demonstrating the 'building blocks' used to identify a final global theme. This paper presents one emergent global theme 'Pivotal Role of the PHN'. Fig. 1 illustrates the three dependent organising themes: relationship building, fostering coping resources, and health promotion and education.

**Table 2:** Examples of codes to basic themes.

**Table 3:** Moving from basic to organising to global themes.

### 5.2 Sample characteristics

The demographic characteristics of PHNs and mothers are shown in Tables 4 and 5, respectively. Specifically, mothers were married and aged 31–40 years. Majority of nurses were highly experienced and working in urban/city locations.

**Table 4:** Demographic characteristics of PHN participants (*n*=19).

Characteristics	Female*n*=19	%
**Current Role**		
**Registered Public Health Nurse**	16	84%
** Assistant Director of Public Health Nursing**	3	16%
**Years qualified as PHN**		
** 0–5**	4	21%
** 6–10**	2	10.5%
** 11–15**	11	58%
** 16–20**	2	10.5%
**Years caring for Child and Maternal Health Caseloads**		
** 0–5**	4	21%
** 6–10**	2	10.5%
** 11–15**	10	53%
** 16–20**	2	10.5%
** 21+**	1	5%
**Caseload Characteristics**		
** Mainly Child and Maternal Health with some General Care**	5	26%
** Even Mix of Child and Maternal Health and General Care**	12	63%
** Management of a Mix of Child and Maternal Health and General Care**	2	10.5%
**Residential Characteristics**		
** Rural**	4	21%
** Urban**	11	58%
** Mainly Rural with Some Urban**	2	10.5%
** Mainly Urban with Some Rural**	2	10.5%
**Socioeconomic Status (SES) as described by PHNs**		
** High SES**	0	0%
** Middle SES**	12	63%
** Low SES**	5	26%
** Mixed Middle and Low SES**	2	10.5%

**Table 5:** Demographic characteristics of Mother participants (*n*=5).

Characteristics	*n*=5	%
Age range (years)		
31–35	2	40%
36–40	3	60%
Marital Status		
Married	5	100%
Single	0	0%
Number of Children		
1	1	20%
2	3	60%
3	1	20%
Current Feeding Type		
Breastfeeding	3	60%
Formula Feeding	2	40%
Length of time Baby was Breastfed		
Never	1	20%
While in the Maternity hospital	1	20%
>3 Months	2	40%
Mothers identified Support from Family		
Yes	4	80%
No	1	20%
Residential Information		
City	2	40%
Town	2	40%
Village	1	20%

### 5.3 Ethical approval

Ethical approval was obtained from the University Institutional Review Board Research Ethics, the Primary Care Research Committee and National Director of Nursing and Midwifery Services. All participants provided voluntary, written informed consent. Focus group participants signed a confidentiality agreement.

## 6 Results

### 6.1 Building relationships

Building a relationship with mothers is ‘*key*’ (Focus Group (FG) 3, Participant (P) 6) and a ‘*very important’* (FG1, P.1) aspect of the first postnatal visit. PHNs emphasised that first impressions are paramount establishing the relationship between mothers and PHNs for future child and maternal health care. PHNs identified that building a relationship with mothers is paramount compared to completing many technical aspects of a visit:

*‘You are developing a relationship. It is the relationship. It doesn't matter what you have done or haven't done (…)’* (FG4, P. 2)

Mothers’ interviews echoed that first impressions are vital. Rachel, a first-time mother, had negative perceptions of what the first postnatal visit entailed. However, the following extract illuminates that the PHN initiated the foundation for a good relationship:

*‘(…) I hear horror stories of people saying, "oh, your house is too clean for someone who has a baby". Or "PHNs make judgement and comments about people when they come in the door" but there was absolutely none of that at all’.* (Rachel, Mother of 1)

PHNs reported that communication with a mother begins when PHNs receive the notification of the birth and specify they would always visit by appointment:

*‘I suppose we visit by appointment. They know we are coming.’* (FG2, P.2)

One mother specifically spoke about the importance of a planned visit:

*‘(…) I think it is much better if you have a planned time and you know somebody is calling instead of just landing on your doorstep (…).’* (Rachel, Mother of 1)

PHNs are mindful of being a visitor in mothers' homes and are sensitive in communicating with families in order not to jeopardise the relationship:

*‘(...) I think there is a huge amount of risk of you just entering somebody's house. (...) So it is quite a significant thing how you communicate.’* (FG4, P.2)

Mothers recalled PHNs' sensitive, collaborative communication skills and acknowledged the opportunity to discuss options as opposed to being dictated too. One mother identified that health professionals often offer conflicting advice; however, the sensitive nature in which the PHN communicated with Rachel, indicated a trusting relationship:

*‘(...) But I suppose I felt the nurse* [PHN] *was more practical in that every baby is different you can't just follow a manual or a book and do everybody the same. (…) so, I suppose I found the information from them* [PHN] *was a lot more useful to me.’* (Rachel, Mother of 1)

Tailoring the visit emerged as necessary for PHNs. They noted that listening to the mother's experience and getting a ‘*clear picture’* (FG2, P.1) of how she is coping with motherhood allows the PHN to provide specific supports:

*‘(...) you are asking her "how was the pregnancy, did you enjoy it? How was the delivery?" (...) you determine very quickly where she is. Is she in a good place? Is she in a bad place? Is she sore? (...) You know if they are feeling very vulnerable or tearful you can ask them just one question that might open up that whole door you know.’* (FG4, P.4)

‘*So, you tailor the information depending on the visits (…)*’ (FG1, P.3)

Mothers confirmed this practice and expressed appreciation:

*‘(…) it could be a bad experience as well you know (...) I thought it was a good thing to ask (...).’* (Eimear, Mother of 2)

*‘(...) and it was very specific to your own circumstance and to your own home and your own family (…) it wasn't just a general talk or anything like that’.* (Marie, Mother of 2)

While this organising theme demonstrates relationship-building stems from the first postnatal visit, mothers noted a lack of trust, limited sensitive or collaborative communication and more of a ‘*technical’* visit in deference to relationship building:

*‘She wasn't here very long, really. I think kind of because she was the third baby she was happy to kind of come in and do what she had to do and go. She has only been the once now. (...) she came in, did what she had to do and left (...)’* (Niamh, Mother of 3)

### 6.2 Fostering coping resources

Empowering mothers through reassurance and support to care for themselves and their infant emerged as a second theme. Mothers spoke of the reassurance from the nurse and appreciated she was ‘*very thorough’* and *‘very particular’* (Eimear, Mother of 2). Irrespective of the number of children they had, mothers felt that of all health professionals, the PHN gave them confidence and provided reassurance of self-belief in their ability to care for their new infant and themselves as depicted from the following accounts:

*‘(...) and it was just helpful to know that things were on track and even though I had another child and I breastfed him, you would forget from previous experiences how you started off (…), just to have somebody to reassure you that you were doing things and you were doing them right'.* (Marie, Mother of 2)

PHNs acknowledged empowering mothers and confirmed they provide information, education, practical hands-on help and availability of their support to give mothers self-confidence in their abilities:

*‘But you are empowering them to be a parent and to be able to connect with somebody that might be able to support them (…).’* (FG4, P.1)

Mothers appreciated verbal and written information, as well as practical advice during the visit and subsequently:

‘*(...) There is a lot to take in but having the leaflets then helps with that. So I was able to check back in the book (...)’*. (Marie, Mother of 2)

Having the PHNs contact details, clinic times, and knowing the PHN would be out to visit again provided a sense of security for mothers –they appreciated access to an accessible and efficient nurse:

*‘So it was just good to have that* [Contact Number], *and it didn't matter how busy it was she did always ring me back. (...) I did actually feel they* [the PHN] *are around the corner (...)’* (Rachel, Mother of 1)

Both PHNs and mothers specified that by giving mothers the opportunity to ask questions is a means of confidence building:

*‘(…) I think you can relieve a lot of anxiety. Often parents have lots of questions and it is just to give them an opportunity to ask those (…).’* (FG2, P.1)

*‘(...) and any questions that you had you could ask them (...) I wanted to ask her questions (...) and she was really able to give me a lot of information.’ (*Marie, Mother of 2)

Speaking out loud during the newborn examination was also a method acknowledged by PHNs as a means of reassuring mothers:

*‘(…) by involving them and saying it out loud you are taking some of the fear away.’* (FG4, P.2)

PHNs identified that they need to use their local knowledge of resources to assist mothers identify and use appropriate resources. PHNs specified that the supports required are very much dependent on the mother's support network. Through advising mothers of available resources, mothers can make links with each other, with local groups and supports. However, it is acknowledged that once information is offered it is up to the mother herself to engage:

*‘Isn't it wonderful that if we are there and we can get these mothers together and we can actually get them to help themselves and we can now step back from because that group is going and they will be friends and it will develop (...)?*’ (FG3, P.6)

*‘Obviously we can only advise and refer and if we want someone to go and do a parenting course or get them some support we can only advise them, we can't force them to take it.’* (FG3, P.5)

PHNs felt strongly that by giving mothers information they need empowers them to have confidence ‘*to look for help when needs be*’ (FG4, P.5):

‘*And to give them the confidence that if the baby is not well to go to the doctor or to go to XX Children's Hospital (…)’* (FG2, P.2)

*‘(…) you would hope they would develop a sense of knowing when there is something wrong or I suppose that is what you are trying to do isn't it.’* (FG4, P.1)

It must be noted that not all PHNs recognised mothers’ needs. Although it illuminated that PHNs offer passive support by advising mothers to contact the public health nursing service, it can be seen from both mothers’ and PHNs’ narratives that mothers may be hesitant in contacting them even when support is required. PHNs recognise this as a gap in the service:

*‘(...) I was more worried I think because she was a girl. (...) You see I was alright because I had other people I could ask if I needed to know anything (...) As I said I felt that she felt I knew what I was doing. (...) [The PHN] gave me her number and said if there was anything I wanted or needed or anything to give her a call.’* (Niamh, Mother of 3)

*‘(...) but obviously we always say our door is always opened and they can contact us if they have any concerns but often times they won't so from that point of view there is a gap isn't there (...)’* (FG3, P.6)

Another mother identified that they appreciated the extra support and being empowered to contact the PHN as required:

*‘So that was really good and reassuring to know that I didn't have to wait a full week for somebody to call out, that I could take him* [to the clinic]*that I could make a phone call anytime I wanted to if I had any questions or problems or anything with the child’.* (Marie, Mother of 2)

### 6.3 Health education and promotion

Health promotion emerged as essential. Mothers recalled receiving information on postnatal depression (PND), and they felt comfortable talking to the PHN about their feelings and coping skills. PHNs acknowledged the importance of discussing postnatal depression and their approaches differed; dependent on the information received from individual maternity hospitals:

*‘(...)some of the hospitals are great, you get a detailed discharge summary, so they may have written down the mother has a history of depression in the past or anxiety or a history of PND (...). Whereas some of the discharges you get nothing (...)’* (FG3, P.5)

PHNs in this study noted ‘*a level of responsibility to ensure a mother is well’* and limit the risk of PND. PHNs reiterated the ‘*key visit’* establishes their role in caring for mothers with PND; some stated mothers may not require subsequent referral to a GP:

*‘(...) some people don't necessarily need to be referred to the GP that often they wanted somebody to sit and talk to, to say what they need to say in a safe environment and that is back again to your key visit where you have developed a relationship with someone (...)’* (FG3, P.5)

Mothers and PHNs recognised child safety was an essential component of the first visit, including information on child protection, sudden infant death precautions and positive parenting:

*‘(...) overcrowding (...) You could have three family units living in a three-bedroom house. (…) So there is no room for three cots in the box room. So, there is co-sleeping so safety awareness, sudden infant death syndrome.’* (FG2, P.1)

Mothers recalled Inconsistences and gaps in the advice provided:

*‘Well they told me in relation to sleep and put her on her back or whatever, but they didn't say anything else. (...)’* (Eimear, Mother of 2)

*‘(...) She spoke about cot death and how to ensure that the room isn't too hot and make sure that the child wasn't too hot and which blankets to use when the baby is going to bed (…).’* (Marie, Mother of 2)

For self-care, mothers recalled advice on wound care. However, not all mothers remembered having their wounds reviewed. One mother recalled that not having her wound examined added to her anxiety:

*‘It was my first section, and she didn't examine my scar or anything. She didn't examine it or me or anything she just asked how it was, and I said, "yeah it is fine", and she just said to keep it clean (...)*’ (Niamh, Mother of 3)

Nonetheless, PHNs identified that to ‘*actually physically look’* at the wound was an imperative component of the first postnatal visit. PHNs reported this was the *‘only chance’* (FG4, P.3) they had to review a wound.

Finally, a range of practical advice was provided to empower mothers. This included information on jaundice, feeding, sleep, ‘*changing the baby's nappy, cord care, eye care, bathing the baby; all of those things that are pertinent to the initial care of a newborn baby'* (FG1, P. 4). All PHNs discussed the practical ‘*newborn examination of the baby, full head to toe examination (...)’* (FG1, P.4) which includes weighing and measuring the baby, ‘*looking at colour, breathing, reflexes, chest movement, fontanel’* (FG4, P.3), elimination, *‘the genitalia, and distension of testes’* (FG4, P.4). PHNs discussed how they would involve the mother in the infant's examination and give them feedback as a means of educating:

*‘But I think quality too. I think when you are doing the baby check. Just say "come on over here now and this is his fontanel, have you felt his fontanel?" "Oh no I didn't want to touch it. Do you want to touch it now? And that closes at 18 months. Another one back here". Bringing them down along the body even to the boys with the boobs. I would say to them "just feel here now. And it's just the hormones and in case you find that later on you might be worried about it. And it will go down". Cord down along, (...) The feet, turn them over look at that back you know talk wees and poos.’* (FG4, P.2)

Weight, length and head circumference measurements were recognised as tasks the PHN carried out. By providing this information they offered reassurance of ‘normal’. They included advice explaining infant behaviours to support mothers through the reality of the postpartum period:

*‘(...) People sometimes want instant baby sleep for three hours, wake feed, and it doesn't happen like that (...)’* (FG3, P.3)

While only one mother recalled advice in relation to jaundice, the importance of advising mothers about the signs and symptoms of jaundice and when to seek medical attention was evident in PHNs' focus groups:

*‘(…) Look at the jaundice, talk about how jaundice comes, how jaundice goes. What the important things are. What are the dangers you know if the baby is sleepy? How is the feeding going? (...) you are empowering them.’* (FG4, P.2)

Support for mothers who breastfeed was essential. PHNs believed that support or lack thereof affected mothers ability to continue breastfeeding. Observing a mother breastfeeding to ensure the baby is latched on and feeding correctly was considered essential to successful breastfeeding:

*'Very much to observe, look at the latch, looking to see if she has a supply- if her milk is in (...).'* (FG3, P.5)

Mothers reiterated this sense of reassurance with being observed breastfeeding. Weighing the infant and weight gain also gave a sense of reassurance to mothers that feeding was going well:

*‘(...) she just had a look how he was latching, just made sure that the latch was good, and also, of course, she checked his weight (…) he had gained weight (…) she assured me that was a really good sign that the breastfeeding was going well.’* (Marie, Mother of 2)

Mothers who formula-fed their infants felt the practical feeding advice did not meet their needs. Mothers articulated that PHNs should not presume that mothers know how to prepare bottles, even if they have previous children. More practical advice, demonstrations and less conflicting advice around formula feeding are required:

*‘So that is a bit confusing definitely, and if it is your first, I would say it is highly confusing. (...) it would be nice to have* [pause] *Even that yeah definitely how you go about your day's bottles (...)’* (Louise, Mother of 2)

*‘I had to feed her every two hours, so I had to ring the nurse then and ask her what to do because I wasn't sure whether I was putting her on the hungrier food, but she suggested no it was totally up to me. (...) I didn't know what to do (...)’* (Niamh, Mother of 3)

PHNs themselves acknowledged reduced supports for mothers who are formula feeding their babies. PHNs stated that while it was against their health promotion roles to provide supports for formula feeding mothers, they did discuss and support mothers:

*‘(…) It is not the young girl that hasn't finished school and is going back to do her leaving cert. It is as much aimed at the doctor, and the teacher and housewife who have everything spot on you know (...) it is aimed at everyone’* (FG3, P.3)

*‘I went to a woman the other day who is in her late 30s, and she thought she made up the formula with regular milk. A professional woman like 37 or 38 who has her own business, and she was shocked that she made it up with water. No idea’* (FG4, P.5)

However, despite both mothers and PHNs identifying individualised care, the practical advice recalled by mothers was limited and did not always appear to meet their needs.

## 7 Discussion

This study identifies both positive care practices and challenges that exist during the first postnatal visit, presenting mothers' satisfaction with the care received, but also a lack of standardisation. The findings have implications for a large population given that there are over 60,000 births annually in Ireland (Central Statistics Office, 2020), and over 730,000 in the UK (Statista, 2020). Mcleish et al. (2019) identified that first-time mothers' satisfaction with postnatal care and their confidence as new mothers were primarily influenced when postnatal needs were met. While mothers in the current study were satisfied with the care generally, discrepancies between priorities for postpartum care exist, particularly practical care. Seeking service user and health professional views provides a greater opportunity to understand the quality of direct care (Schmied and Bick, 2014, Hasson et al., 2019).

### 7.1 Lack of standardised care

Lack of standardised practice was a consistent thread running through the current study. Much of the care provided was inconsistent. This study highlights that mothers often receive conflicting advice from health professionals, corroborating findings of earlier studies (Newburn, 2010, Razurel et al., 2011, Barimani and Vikstrom, 2015). PHNs in the current study identified that they ‘tailored’ care to meet mothers’ needs based on observations at the initial postnatal visit. While Schmied (2014) identifies that mothers’ priorities may differ, Cheng et al. (2006) suggests that mothers’ and nurses’ priorities for postpartum care and education may differ, and Cowley et al. (2018) also allude to this recently. Differing priorities emerged during mothers’ interviews in the current study as they revealed that PHNs did not always meet their needs. Phelan (2014) and Monsen (2017) both identify that care can be tailored to suit individual needs. However, they both acknowledge that there are ‘core areas’ of care that must be discussed as mothers may be hesitant asking for help or asking to be shown due to fear of being judged (Phelan, 2014, Wayman, 2019). Dahlberg et al.’s (2016) qualitative study identified that mothers are vulnerable during the early postnatal period describing feelings of emotional instability and insecurities, thus explaining why mothers may be hesitant in asking for help and reiterating the importance of providing standardised care, tailoring the information to address the understanding of each mother. A tailored approach to care reliant on an assessment of a mother's knowledge and circumstances emerged as an essential practice in Ireland, and these findings are similar globally (Monsen et al. 2017, Mcleish et al. 2019).

The challenges with communication from maternity care to the community are highlighted within the current study. Unlike many countries globally (Riordan et al., 2015, Essen et al., 2018), no electronic records or individual patient identifier system exists currently in Irish healthcare. Nurses in the current study highlighted difficulties in obtaining complete information from maternity services to guide postnatal visits, and these findings support Phelan's (2014) earlier conclusions. Within the current study, discourses indicating inconsistency and lack of standardisation in care emerged such as the physical care of the mother, infant feeding practices and child safety, and points to a need for ongoing educational development for PHNs in relation to core areas of care. For example, despite the increase in caesarean sections and third-degree perineal tears (Callaghan et al., 2010, Dahlen et al. 2013), the reported care experiences in the current study were inconsistent and wounds were not being observed at the first postnatal visit. This is consistent with national and international discourse (Haran et al., 2014, Higgins et al., 2017, Higgins et al., 2018).

### 7.2 Relationship-building

The current study identified that first impressions, relationship-building and collaborative communication are paramount for establishing a trusting relationship. Findings from the current study corroborate the body of literature noting PHNs use of listening, observing and talking skills are intended to help mothers engage with the public health nursing service making it possible to open up the discussion and expose any concerns mothers may have (Appleton and Cowley, 2008, Cowley et al., 2013). The PHNs in this study afforded time to listen to mothers’ experiences and requirements in order to provide individualised care; the essence of a trusting mother-PHN relationship (Appleton and Cowley, 2008, Leahy Warren, 2005, O'Dwyer,2009, HSE, 2013, Bidmead et al., 2016)

The findings in this study are consistent with Cowley et al. (2013) who identified the importance of relationship-building in home visiting and should start at the first contact. Relationship-building is complex and requires PHNs to have excellent communication skills, provision of feedback on practices and ongoing professional development (Aston et al., 2015).

The current study identifies that PHNs emphasise the importance of sensitive communication to reduce parental anxiety, and these findings are consistent with earlier studies (HSE, 2005, Leahy-Warren, 2005, Cowley et al., 2013). PHNs’ verbal and non-verbal communication skills are intended to help mothers’ engagement, making it possible to open up discussions and build trust (Appleton and Cowley, 2008, Cowley et al., 2013). First impressions are vital; Cowley et al. (2015) found that a productive mother-PHN relationship forms over repeated contacts, and mothers are more likely to seek help from their PHN with whom they have an established relationship. Finally, the importance of communicating practical advice for self-care and infant care were reported and support previous evidence from Leahy-Warren (2005) and Cowley et al. (2013).

### 7.3 Empowering mothers

Maternal confidence is a mother's perception of her ability to care for her infant (Badr, 2005). Mothers and PHNs in this study identified that observing mothers carrying out essential care for their newborn or providing demonstrations was empowering, and is consistent with Phelan's (2014) and Kurth et al.’s (2016) published studies.

Similarly to the findings of the current study, interactions, encouragement and information giving from PHNs, supporting mothers’ decision-making to recognise early signs of health problems, have been shown to relieve mothers’ nervousness, thus providing reassurance, self-confidence and self-efficacy with their parental role (Phelan, 2014, Kurth et al., 2016). The health education and promotion discussed within the current study's findings identifies the requirements of mothers and what is acknowledged as essential health promotion advice provided by PHNs. Without this advice the quality of the care provided at the first postnatal visit would be questioned. This advice ‘sets a mother up’ and empowers her to care for herself and her new infant. However, while PHNs in the current study endeavoured to assure mothers in their caring role, the mothers contradicted this, highlighting a gap and a requirement for more advice and information to access and use after visits. In this study, mothers recalled receiving conflicting information from different health professionals, adding to their anxieties. However, PHNs were praised for providing the most consistent and sensitive advice to mothers. Providing consistent information and recording evidence of this assist in continuity of care (Newburn, 2010, Razurel et al., 2011, Gao et al., 2012, Ong et al., 2014, Schmied et al., 2013, Barimani and Vikstrom, 2015, Kurth et al., 2016).

The current study identified that supporting mothers by reviewing the risk of postnatal depression is critical at the first postnatal visit. All mothers recalled being provided with information in relation to PND and this finding is consistent with previous studies (Cooper et al., 2003, Turner, 2010). Cooper et al. (2003) identified mothers found PHNs to be more effective than other health professionals in providing home based interventions for PND. However, as with many other studies the current study focussed on PND to the exclusion of the wider spectrum of psychological health (Jomeen et al., 2013, Highet et al., 2014, Noonan et al., 2016). This may prevent women from identifying other mental health problems and seeking appropriate treatment (Jomeen et al., 2013, Highet et al., 2014, Noonan et al., 2016). This study corroborate study findings by Noonan et al. (2016) and Mc Loughlin (2018) that PHNs require support structures to care appropriately for women with psychological issues. Higgins et al. (2018) identified educational needs of PHNs in caring for women with mental health issues including but beyond postnatal depression is required. A starting point in Ireland for PHNs would be to train PHNs in the use of listening visits. Internationally, listening visits by PHNs are widely used as an effective treatment for PND (Cooper et al., 2003, Turner, 2010). A step in the right direction in the UK will be rolled out in 2020 as mothers will receive a mental health assessment together with a physical assessment at six weeks postnatally by their family doctor (Campbell, 2020).

Health promotion is an essential role of the PHN in encouraging better health and PHNs are ideally placed in the community to offer this service (Irish Nurses and Midwives Organisation, 2013). Promoting breastfeeding is a key health promotion role of the PHN and this has been acknowledged in the current study. This study highlighted the support provided to breastfeeding mothers, however support for mothers who were formula feeding their infants were limited. It illuminated that PHNs felt it was ‘against their health promotion role’ yet it was clear that mothers needed this support and advice in order to ensure safe feeding practices for their infants and thus should be a core care area discussed and demonstrated at the first postnatal visit.

This study highlighted the quality of public health nursing practice at the first postnatal visit and identified quality improvement requirements for care provided to mothers and their infants by PHNs. Core areas of care and deficits in practice have been identified by both PHNs and mothers. Standardising core areas of care is arguably a means of improving the quality of care provided to mothers during this visit.

### 7.4 Limitations

This qualitative study and findings represent the views of those who participated and may not be representative of all nurses or mothers. Recruitment of mothers was limited to one geographical area and five mothers, all of whom were married within the middle socioeconomic class, participated. No data were collected from mothers who were single, in a lower socioeconomic class or women in the Black, Asian and Minority Ethnic (BAME) community. Despite all attempts to access wider groups of mothers for inclusion in the study, it was not possible.

The inclusion of single mothers or those in the lower socioeconomic class would have provided a voice to marginalised groups. Data obtained provide honest accounts from mothers who participated and identifies their challenges.

The use of focus groups may have been a barrier to encouraging nurses participation. However, this method provided rich data from the interactions between group members.

## 8 Conclusion

This is the first study of nurses and mothers in Ireland identifying care provided at the first postnatal visit. Few other recent studies internationally have combined health professional perspectives and mothers’ experiences in the postpartum period. A standardised routine ‘core care’ guideline for women and their babies exists in the UK; however, this study identifies a lack of standardised practice and lack of postnatal guidance for PHNs which is similar in many countries worldwide (Haran et al., 2014). While NICE guidance exists, this is the first Irish study to present mothers' experiences of care delivered at a first postnatal visit. This qualitative study illuminates mothers’ and PHNs views and experiences, and can assist policymakers to identify core areas of care to be included in a national guideline including for example communication guidelines, specific health promotion education for mothers and infants, physical assessments of mothers and infants and means of establishing support mechanisms for mothers. Together with policy development, nurses require professional education programmes in relation to these core areas of care to ensure evidence-based, high quality, safe and individualised care.

## Declaration of Competing Interest

No conflict of interest has been declared by the authors.

## Appendix Supplementary materials

Image, application 1
