# Supplementary material for: Identifying the role of public health nurses during first postnatal visits: Experiences of mothers and public health nurses in Ireland
Source: Int J Nurs Stud Adv. 2021 Jan 5;3:100017. doi: 10.1016/j.ijnsa.2020.100017 (PMC11080328; doi:10.1016/j.ijnsa.2020.100017)
Supplement: Supplementary file 1 [file mmc1.docx]

### **Appendix 1 Focus Group topic guide with Public Health Nurses**

**Briefing**

Introduction of the Researcher

Study Explained

Participants informed about anonymity, confidentiality and recording of the focus group interview.

Participants asked that one person speaks at a time for recording purposes.

Questions invited from participants.

Date: Venue: Time:

1. May I begin by asking you to tell me about your role as a Public Health Nurse in relation to child and maternal health?
2. Can you tell me about quality care during the primary visit?
3. Can you give me examples of quality nursing care or experiences of quality nursing care?
4. Can you recall any incident where you could not provide quality care?
5. What aspects of care would you consider the most important for the primary visit and postnatal check?
6. What can you tell me about Key Performance Indicators?
7. What can you tell me about Quality Care-Metrics?
8. Can you tell me about your views of the use of QCMs in PHN practice?
9. Is there anything else you would like add?

**Debriefing**

Do you have any questions you would like to ask?

Thank you for your time and participation in this study.

Probes

Can you think of any examples or stories that explain it?

You mentioned_______________ what do you mean by that?

Have you had personal experience of that?

### **Appendix 2 Interview Schedule for New Mothers**

**Briefing**

Introduction of the Researcher

Study Explained

Participant informed about anonymity, confidentiality and recording of the interview.

Questions invited from participant.

Date: Venue: Time:

1. Introductions
2. Can you tell me about your experience with the PHN the first time you met him/her following the birth of your baby?
3. What aspects of your experience would you consider high quality care provided by the PHN during the primary visit?
4. Was the experience as you expected?
5. What information was given to you?
6. Did you find this information the PHN provided beneficial and of high quality?
7. If there are other mothers you think would be interested in talking to me can you give them my contact details please?
8. Is there anything else you would like to add?

**Debriefing**

Do you have any questions you would like to ask?

Thank you for your time and participation in this study.

**Probes**

Can you think of any examples or stories that explain it?

You mentioned_______________ what do you mean by that?

Have you had personal experience of that?
